# Optothermal Needle‐Free Injection of Vaterite Nanocapsules

**DOI:** 10.1002/advs.202305202

**Published:** 2023-12-03

**Authors:** Denis Kislov, Daniel Ofer, Andrey Machnev, Hani Barhom, Vjaceslavs Bobrovs, Alexander Shalin, Pavel Ginzburg

**Affiliations:** ^1^ Center for Photonics and 2D Materials Moscow Institute of Physics and Technology Dolgoprudny 141700 Russia; ^2^ Department of Electrical Engineering Tel Aviv University Ramat Aviv Tel Aviv 69978 Israel; ^3^ Light‐Matter Interaction Centre Tel Aviv University Tel Aviv 69978 Israel; ^4^ Triangle Regional Research and Development Center Kfar Qara 3007500 Israel; ^5^ Institute of Telecommunications Riga Technical University Riga 1048 Latvia; ^6^ Faculty of Physics M. V. Lomonosov Moscow State University Moscow 119991 Russia

**Keywords:** femtosecond laser pulse, needle‐free injection, thermal expansion, Van der Waals adhesion force, vaterite nanocapsule

## Abstract

The propulsion and acceleration of nanoparticles with light have both fundamental and applied significance across many disciplines. Needle‐free injection of biomedical nano cargoes into living tissues is among the examples. Here a new physical mechanism of laser‐induced particle acceleration is explored, based on abnormal optothermal expansion of mesoporous vaterite cargoes. Vaterite nanoparticles, a metastable form of calcium carbonate, are placed on a substrate, underneath a target phantom, and accelerated toward it with the aid of a short femtosecond laser pulse. Light absorption followed by picosecond‐scale thermal expansion is shown to elevate the particle's center of mass thus causing acceleration. It is shown that a 2 µm size vaterite particle, being illuminated with 0.5 W average power 100 fsec IR laser, is capable to overcome van der Waals attraction and acquire 15m sec^−1^ velocity. The demonstrated optothermal laser‐driven needle‐free injection into a phantom layer *and Xenopus oocyte* in vitro promotes the further development of light‐responsive nanocapsules, which can be equipped with additional optical and biomedical functions for delivery, monitoring, and controllable biomedical dosage to name a few.

## Graphical abstract

The concept of needle‐free nanoparticle injection ‐ nanocargoes on a substrate are accelerated with a short femtosecond laser towards a target tissue. The work demonstrates the optothermal laser needle‐free injection of vaterite capsules into the phantom layer and Xenopus oocyte in vitro. These capsules can also be equipped with additional optical and biomedical functions for controlled drug delivery and monitoring.

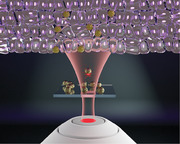

## 1 Introduction

Needle‐free injection of biomedical cargoes into living tissues allows for reducing possible contamination and suppresses inflating unnecessary pain in patients. While quite a few different proposals including laser‐based solutions have been reported,^[^ ^1^, ^2^ ^]^ controllable injection of a single biomedical nanocargo remains a challenge. Here we will explore the capabilities of light‐driven tools to address this challenge. Since the first demonstration by Ashkin in 1970,^[^ ^3^ ^]^ optomechanical tools were significantly advanced and taken toward practical applications,^[^ ^4^ ^]^ where propulsion, acceleration, and trapping of nanoparticles with light are of use.^[^ ^5^ ^]^ Optical tweezers have been found beneficial in biological studies, as they allow controlling the motion of micro‐ and nano‐scale objects, unfolding proteins and molecules, measuring pico‐Newton scale forces, and can grant many other capabilities.^[^ ^6^, ^7^, ^8^ ^]^

Optical forces, acting on isolated systems, are quite well understood and have been assessed in many experiments. In terms of electromagnetic theory, Maxwell's stress tensor linking classical light‐matter interactions with Newtonian forces provides a comprehensive description of the phenomenon.^[^ ^9^ ^]^ The formalism can be further extended to cavity quantum optomechanical regimes,^[^ ^10^ ^]^ where many remarkable effects can be observed, though demanding isolation from an environment, e.g., by cryogenic cooling and high vacuum.^[^ ^11^, ^12^ ^]^ In room‐temperature biological scenarios, however, a range of different physical mechanisms can contribute to interactions. In many cases, thermal forces, emerging from environmental interactions, can prevail over optical and, in fact, govern the dynamics. One of the key contributing effects affecting the optical manipulation of nano‐ and micro‐objects is thermal noise.^[^ ^13^, ^14^ ^]^ Fluctuations affect particle‐environment interactions,^[^ ^15^, ^16^ ^]^ imposing additional constraints on optical tweezing in biological media.^[^ ^17^ ^]^ Furthermore, temperature gradients in an embedding fluid give rise to directional thermophoretic forces acting on small particles. Apart from external non‐isothermal conditions, those gradients can be locally induced by light absorption.^[^ ^18^, ^19^ ^]^ The optically‐controlled thermophoretic effect can be utilized for propelling nano‐ and micro‐objects in fluids and can predominate over purely electromagnetic optomechanical forces.^[^ ^20^, ^21^ ^]^ For example, local laser cooling of a substrate led to colloidal particles and molecules trapping in a low‐temperature region – the so‐called – opto‐refrigerative tweezers.^[^ ^22^ ^]^ Colloidal objects can also be heated directly with a laser, thus inspiring self‐thermophoresis. Nonuniform heating of an absorbing particle, caused by a nonsymmetric illumination, inspires strong self‐thermophoretic forces, e.g.^[^ ^23^, ^24^ ^]^ The symmetry in heating can be broken by an object's design. So‐called Janus particles are typically realized as transparent glass spheres, half‐coated with strongly absorbing gold.^[^ ^25^, ^26^, ^27^ ^]^ Optothermal manipulation schemes were further explored for capturing and separating particles.^[^ ^28^, ^29^, ^30^ ^]^ Relying on the above, understanding optothermal effects is critical to developing advanced tools for tailoring the mechanical motion of nano‐ and micro‐objects iterating with an environment.

An appealing approach to investigate temperature effects in optomechanical scenarios is to compare trapping with monochromatic (continuous wave, CW) and pulsed (e.g., femtosecond) lasers, both having the same average power. Several experimental reports, e.g.,^[^ ^31^, ^32^ ^]^ explored this scenario, and no qualitative differences in optomechanical behavior have been found, apart from strong optical nonlinearities, inspired by high peak powers of femtosecond sources.^[^ ^33^, ^34^, ^35^ ^]^ Since those experiments have been performed in a liquid environment, overdamped mechanical motion and heat diffusion average the impact of short pulses over a repetition rate of a source. To factor out the fluid effects, trapping can be performed in a vacuum, e.g.^[^ ^36^, ^37^ ^]^ However, the studies rarely consider mechanical deformations of particles themselves, at least as being the major effect, which governs the interaction.

**Figure 1:**
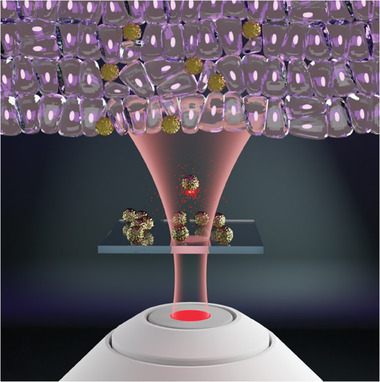
The concept of needle‐free nanoparticle injection – nanocargoes on a substrate are accelerated with a short femtosecond laser toward a target tissue. Optothermal expansion is the underlying mechanism, which governs the light‐particle interaction.

Here we demonstrate a new optomechanical effect, exploring the interaction with short intense laser pulses with particles on a substrate (**Figure** **1**). Observation of the effect requires meeting several conditions on the particle and optical arrangement, as it will become evident hereinafter. The practical outlook on the effect is the needle‐free injection, where a drug cargo is accelerated with light toward tissue and penetrates it.

The manuscript is organized as follows. First, the basic experiment on particle acceleration is demonstrated and release velocities are assessed. After the effect demonstration, basic physical mechanisms, which might contribute to the interaction are discussed. To figure out which one is indeed the most contributing, an additional set of experimental investigations is performed. Finally, particle penetration capabilities are assessed on pathways toward a new needle‐free injection methodology. As a proof of concept, the new thermo‐optical injection mechanism was demonstrated by injecting vaterite cargoes into *Xenopus oocyte (*pre‐stage of a frog egg) in vitro.

## 2 Results and Discussion

### 2.1 Observation of the Particle's Jump

**Figure 2:**
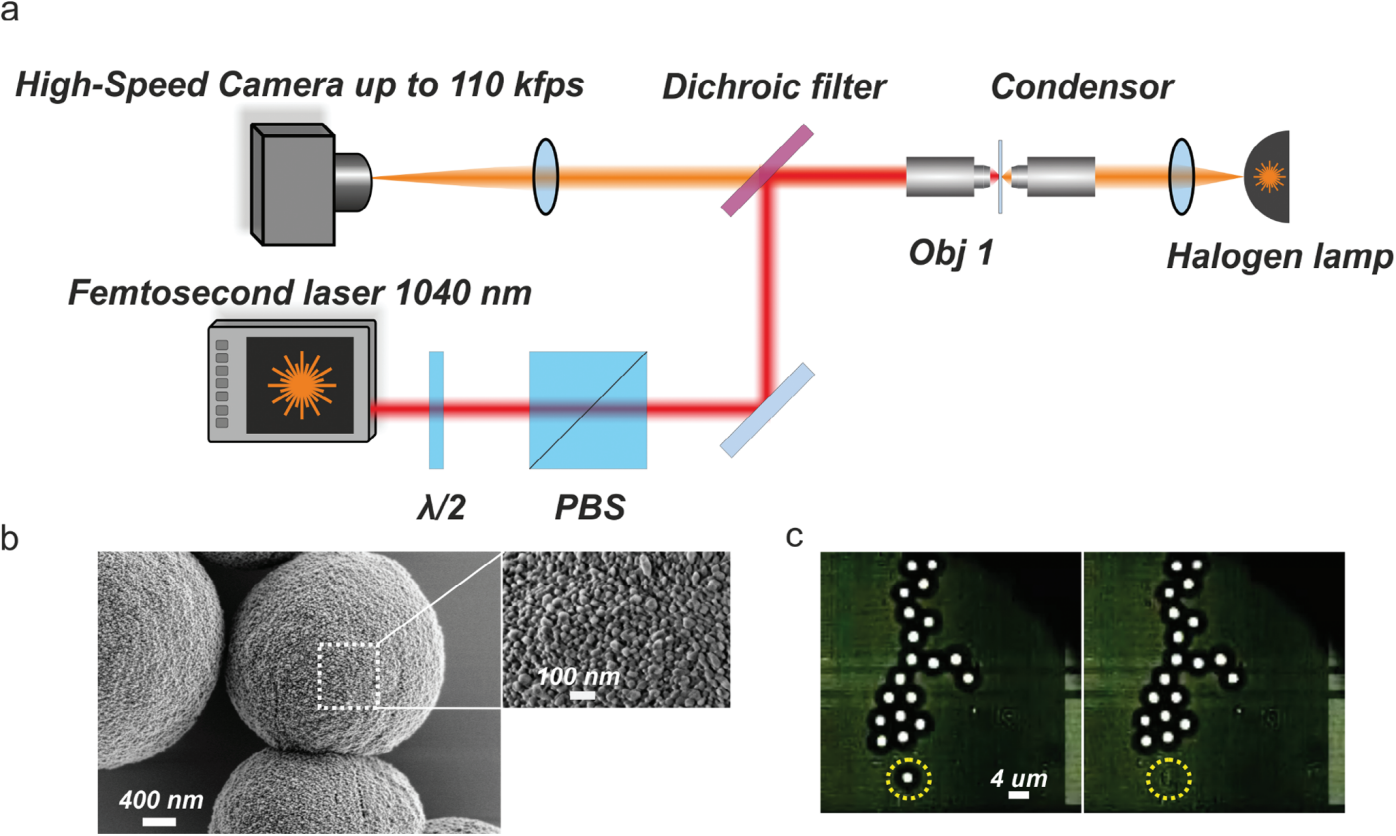
a) Schematics of the experimental setup. b) SEM images of vaterite particles. c) Microscope images of vaterite particles on a glass substrate. Left – before interaction with the femtosecond laser. Right – after the interaction, demonstrating one particle missing.

The experiment was performed with 2 µm radius vaterite nanoparticles, **Figure** **2**b. Vaterite is a metastable polymorph of calcium carbonate (CaCO_3_^[^ ^38^, ^39^ ^]^). Owing to its high load capacity, chemical non‐specific loading, biodegradability, biocompatibility,^[^ ^40^ ^]^ and facile low‐cost fabrication, vaterite nanoparticles are an extremely promising nonorganic platform for drug delivery applications.^[^ ^41^, ^42^, ^43^ ^]^ Furthermore, those cargoes can be designed in different shapes, which affects their optical and biological functions.^[^ ^44^ ^]^ Mesoporous particles can be also loaded with contrast inclusions, e.g., metal nanoseeds,^[^ ^45^ ^]^ which severely affects the light‐matter interaction strength of the composite, as an outlook on future optomechanical manipulation schemes.

To assess the laser capabilities to accelerate nanoparticles, the following experiment was performed. Vaterite nanocapsules were placed on a glass coverslip and positioned on an inverted microscope (Figure 2a). The particles were irradiated with femtosecond laser pulses. A high‐resolution scanning electron microscope (SEM) image of vaterite appears in Figure 2b, highlighting surface roughnesses of 2 um‐size spheres. While particles of those sizes were fabricated for better visualization purposes, smaller dimensions (below ≈0.5 µm) are favorable for drug delivery applications owing to cellular uptake aspects. Figure 2c demonstrates a microscope image of the particles before and after the interaction with the laser, after which one item disappeared. The laser was focused on this exact spot. The minimum speed of particles was estimated from the depth of field of the objective (≈0.8 µm) and the frame rate. The particles were too fast to capture their trajectory even at the highest frame rate of the camera. Consequently, only a lower bound on the velocities can be extracted from the experimental evidence. Since a particle disappeared from the field of view before the next image is captured, the minimal velocity is bounded from below by $\approx 22\frac{cm}{s}$, which will be demonstrated hereinafter as a very underestimating.

To reveal whether the short pulses are responsible for the particle jump, a reference measurement has been performed. The femtosecond laser was replaced by a CW source. Since the jump of particles has not been observed under the CW illumination, the effect is unambiguously related to the high peak power, and a nonlinear time‐dependent phenomenon is involved.

### 2.2 Van der Waals Forces

There are several parameters, which govern the interaction between the particle on the substrate and laser light. The first is the contact potential, which is responsible for sticking the particle to the substrate. In the air environment, Van der Waals forces are the key mechanism, which will dictate the laser power required for the jump. The second factor to reveal is the nature of the force, transferred from the laser beam to the particle. Hereinafter, we will provide a detailed analysis and discussion on the interaction nature. In particular, laser heating of vaterite particles and their fast thermal expansion will be proved to play a key role.

Van der Waals forces bind a nanoparticle and a surface owing to intermolecular attraction. Since this interaction is extremely short‐range, the laser‐induced particle's jump has a threshold effect. In case of a short pulse illumination and low repetition rate, the particle will either remain on the surface or jump, depending on whether the energy in a pulse is sufficient. The threshold condition is given by:

(1) $$\frac{mu_{\min}^{2}}{2}-U_{vdW}=0$$

where the minimal kinetic energy of a particle is equal to the van der Waals potential. Relying on the above discussion, the condition can be approximated as local, i.e., after the particle leaves the surface, the Van der Waals potential can be neglected. In the case of a smooth sphere on a flat surface, van der Waals potential is given by:^[^ ^46^ ^]^

(2) $$U_{vdW}\left(z\right)=-\frac{A_{H}R}{6z}$$

where *A_H_* is the Hamaker constant encapsulating the material properties, *R* is the particle radius, *z* > 0 is the distance between the substrate and the particle surface. The calculation of the Hamaker constant appears in Supporting Information S1. Considering experiments done at normal conditions, as one reported here, a layer of water is always present in the contact area. Consequently, the distance between the particle and substrate surface can be approximated as *z* = 0.3*nm*, corresponding to the size of a water molecule. This number can be also related to the particle's surface roughness (Figure 2b). As a result, the force between a spherical R = 2 µm vaterite and a flat glass substrate is $F_{vdW}^{Vat}\approx -0.34\mu N\;$. In this case, the gravity force*F_g_* ≈ −1*pN* can be neglected.

Following Equations 1 and 2, the threshold velocity is given by:

(3) $$u_{\min}=\frac{1}{2R}\sqrt{\frac{A_{H}}{\pi z_{0}\rho }}$$

leading to uminVat≈5cm/cmss. Considering the scenario of a single pulse incidence, the physical meaning of this velocity is what the particle obtains whether the contact potential was zero. However, in case of a high repetition rate of 100 MHz (given the field of view of the microscope and cm sec^−1^‐scale velocity estimate), the detached particle is hit by at least one more consecutive pulse, providing it with additional kinetic energy. Consequently, the experimental lower estimate and the theoretical prediction of the velocity, based on Equations 1 and 2, correlate well with each other.

Hereinafter, the momentum transfer mechanism will be discussed and the one, responsible for the effect, will be identified.

### 2.3 Light Momentum Transfer Mechanisms

**Figure 3:**
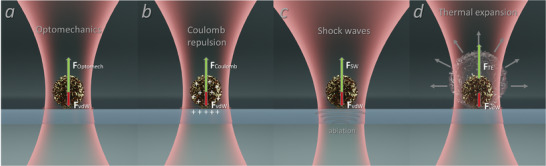
Schematics of four probable mechanisms, which can govern a laser‐induced microparticle jump from the substrate: a) Optomechanical force; b) Coulomb force; c) Material shock waves; d) Optothermal expansion.

Light‐matter interaction phenomena involve many physical mechanisms, especially if nanostructures are involved. After performing experimental studies with several controlled parameters, four main probable mechanisms of light momentum transfer to the particle have been identified and summarized in **Figure** **3**, following reports of, e.g.^[^ ^47^, ^48^, ^49^ ^]^

The first one (Figure 3a) is an optomechanical interaction,^[^ ^50^ ^]^ where light momentum is transferred to a particle via a coherent excitation of polarization in a material, leading to the emergence of macroscopic Lorentz force. Maxwell stress tensor is a tool, which is typically used for analysis. Optomechanically‐driven particle jumps from substrate scenarios have been addressed theoretically^[^ ^51^, ^52^, ^53^ ^]^ and experimentally,^[^ ^54^, ^55^ ^]^ considering intense laser pulses of at least several nanoseconds in duration. However, several special conditions have been met, which are not satisfied in our case. To reveal that this optomechanical mechanism cannot contribute to the observed effect, we have performed a numerical analysis, where optical forces were calculated in the time domain. We show that laser powers, used in the experiment, are insufficient to detach the particle from the surface. A detailed analysis of optical forces appears in Supporting Information S2.

The second probable mechanism is a Coulomb force, which emerges owing to an ultrafast charging of interacting materials due to a multiphoton ionization (Figure 3b).^[^ ^56^, ^57^ ^]^ Charges are created on both the substrate and the particle, resulting in a repulsion. The third hypothesis is “shock waves” (Figure 3c).^[^ ^58^, ^59^, ^60^, ^61^, ^62^ ^]^ Those are caused by a local ablation of a glass substrate, followed by a physical extraction of matter. In this case, the light should be focused on the substrate underneath the particle to cause a significant interaction. Both of those effects share similar underlying physical principles. The key mechanism is multi‐photon absorption followed by ionization, which is a nonlinear process requiring high peak powers of an excitation source. Calcite has E_g_ = 6 eV, which means that at least 5 photons are required for a non‐linear multiphoton ionization caused by a 1040 nm wavelength laser. However, the probability of such a process is negligible. The fluence in our experiment is ≈0.15 J cm^−2^, which is insufficient to make this process dominant. Also, the substrate was not damaged during the experiment, indicating that there is no matter extraction. This confirms that Coulomb repulsion and shock waves are not the main mechanisms of the laser‐induced jump of particles.

The fourth mechanism is a thermal expansion (Figure 3d).^[^ ^63^, ^64^ ^]^ Light absorption causes the material expansion and, owing to the substrate‐induced broken symmetry, a fast elevation of the particle's center of mass occurs. Hereinafter, we will analyze this mechanism and demonstrate that it is indeed the one, responsible for the particle's jump.

### 2.4 Momentum Transfer by Thermal Expansion

Vaterite microcapsules have a relatively weak linear absorption, which was experimentally demonstrated in^[^ ^65^, ^66^, ^67^ ^]^ without specification of the mechanism. The absorbed energy leads to a fast heating of the particle and, as a consequence, thermal expansion – reversible deformations of the lattice. Thermal effects can lead to the appearance of internal stresses in a microparticle, which manifest themselves as vibrations and expansions of the form factor.^[^ ^63^ ^]^ Similar effects were observed considering other scenarios, e.g., the non‐stationary displacements of the air‐gold interface under the action of femtosecond laser radiation.^[^ ^68^, ^69^ ^]^ The experimental technique was based on the probing beam deflection from the surface and demonstrated thermal expansion rise times of ∼100 ps. The effect was attributed to the nonequilibrium diffusion and thermalization of photoexcited electrons.

**Figure 4:**
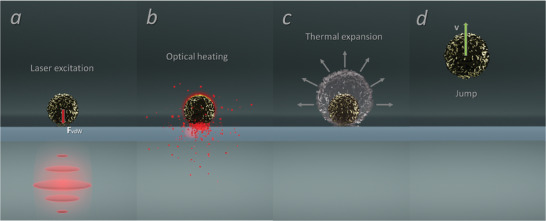
Stages of the optothermal jump – a) laser excitation, b) light absorption, c) thermal expansion, and d) jump. The processes a – d) happen in parallel within a picosecond time scale.

In our arrangement, the fast deformation of a particle leads to a shift in its center of mass respectively to the surface, leading to the particle's jump. The stages of the process are shown in **Figure** **4**. Hereinafter, we will estimate the heating rate of a vaterite microparticle, considering its thermodynamic parameters and laser radiation intensity. Conditions, which are required for the particle's jump, will be estimated.

In the case of short pulses, non‐equilibrium phenomena may occur, nevertheless, the one‐temperature model (OTM), being a simple approximation for the initial estimate,^[^ ^70^, ^71^, ^72^, ^73^, ^74^ ^]^ was found sufficient (see Sections S3 and S4, Supporting Information for the details). For pulse durations lesser than 100 ps, the particle can be approximated as isolated since the heat removal by embedding air occurs on larger timescales. Consequently, the heat equation for the system is:

(4) $$\rho C\frac{\partial T}{\partial t}=q\left(r,t\right)$$

where 𝑇 is the particle's temperature; 𝐶, 𝜌 — specific heat capacity, mass density; $q\left(r,t\right)=q_{0}\exp\left[-\frac{2{\left(t-t_{0}\right)}^{2}}{\tau^{2}}\right]$– is the volume density of the absorbed electromagnetic power, where q_0_ is a constant, which depends on the material absorption and the laser intensity (Section S4, Supporting Information for the details). We also assume uniform heating of a nonresonant small particle. The absorbed power resembles the time envelope of the laser pulse (assumed to be Gaussian with the width τ = 100*fs*).

Equation 4 has an analytical solution, which can be used to estimate the maximal temperature of the particle by taking the limit (*t* → ∞). The time here is longer than the pulse duration and transients inside the particle and shorter than the heat outflow to the air:

(5) $$T_{MAX}^{OTM}=T_{0}+\frac{\sqrt{\pi }{\overset{︷}{\tau q_{0}}}^{const}}{2\sqrt{2}\rho C}\left[1+erf\left[2\sqrt{2}\right]\right]=T_{0}+▵T_{MAX}^{OTM}$$

where $erfx=\frac{2}{\sqrt{\pi }}\int_{0}^{x}e^{-\xi^{2}}d\xi$ is error function and T_0_ is the ambient (room) temperature. The upper estimate for the center of mass velocity *u* of the jumping the particle is:

(6) $$u=\frac{dR}{dt}=\alpha \frac{dT}{dt}R_{0}$$

where α‐ the material thermal expansion coefficient [*K* ^−1^], and *R*_0_ – particle's radius before interaction with the laser. Vaterite spherulite structure contains single nanocrystal subunits that are arranged as a bundle of fibers tied together at the center and spread out at the ends (so‐called “heap of wheat” model ^[^ ^75^ ^]^). While vaterite has a non‐diagonal thermal expansion tensor with position‐dependent components, we will use an experimentally verified isotropic averaged value, corresponding to calcite $\alpha_{zz}^{C}=22.6\cdot 10^{-6}\;K^{-1}$,^[^ ^76^, ^77^, ^78^ ^]^ as a mere approximation. The estimated velocity acquired by a particle upon repulsion from the substrate as a result of thermal expansion is then given by:

(7) $$u=\frac{\alpha_{zz}^{C}▵T_{MAX}^{OTM}R_{0}}{\tau }$$

The detachment of a particle from the surface is of a threshold nature. If the linear momentum acquired by the particle as a result of the thermal expansion is sufficient to overcome the Van der Waals potential energy, then the particle jumps from the substrate with a nonzero velocity, otherwise, it remains bound:

(8) $$v_{z}=\left\{\begin{array}{cc} \frac{\alpha_{zz}^{C}▵T_{MAX}^{OTM}R_{0}}{\tau }, & u\geq u_{\min}\\ 0, & u<u_{\min} \end{array}\right.$$

**Figure 5:**
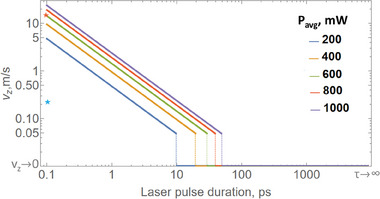
Particle's velocity as a function of laser pulse duration and average incident power (Equation 8). Blue star marker – v ≈ 22 cm sec^‐1^ experimentally obtained a lower bound estimate (Section 2). Red star marker – v ≈ 15 msec^‐1^ a tighter bound assessed by a ballistic experiment (Section 4).

Equation 8 allows estimating the conditions required for the particle jump. **Figure** **5** demonstrates the velocity as a function of the pulse duration for several powers. Introducing the parameters of the experiment, we found that the velocities can be as high as tens of m sec^−1^. This result does not contradict the initial experiment, as it solely provided the lower bound of 22 cm sec^−1^, which is now found to be not tight. m sec^−1^ scale velocities suggest assessing the capabilities of particles to penetrate into a phantom sample, which will be done next.

### 2.5 Temperature of Vaterite Nanocapsules

**Figure 6:**
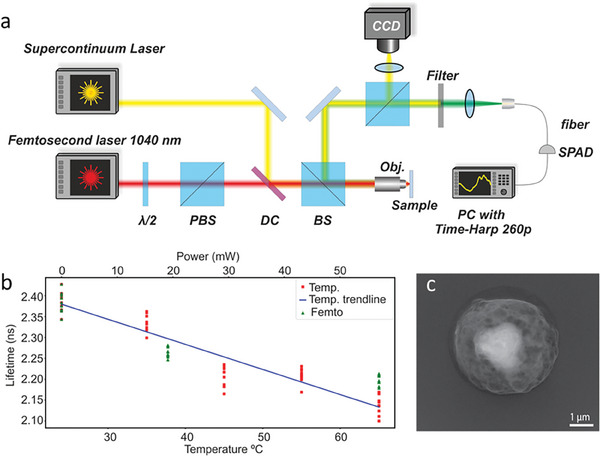
a) Schematics of a setup for particle temperature measurements with fluorescent lifetime. b) Lifetime‐based particle thermometry with Rhodamine B, infused into vaterite. Red dots – reference lifetime and a function of the environmental temperature (lower x‐axis). Green dots – lifetime as the function of an averaged laser poser (upper x‐axis). Blue solid line – trendline. Each x‐axis point data was measured for 10 particles. c) SEM image of a 2um vaterite, stuck in SU8 polymer layer after the optothermal jump.

In order to ascertain the temperature induced in the particles upon illumination with femtosecond pulses we used fluorescence lifetime‐based thermometry, e.g.^[^ ^79^, ^80^ ^]^ The scheme of the experimental setup is shown in **Figure** **6**a.

Figure 6b is the lifetime versus the environmental temperature. Red dots correspond to the reference measurement – the sample was heated to a temperature (lower x‐axis on the plot) and the lifetime was retrieved. The next step is to illuminate the particles with the femtosecond laser. The averaged power is the upper x‐axis on the plot. The blue solid curve is the trendline. The graph allows for relating the laser power to the particle's temperature by assessing x‐axes versus each other. Since this temperature measurement corresponds to time averages and steady‐state conditions, the process can be described with the heat diffusion equation, where the source is a CW illumination. The steady‐state temperature is given by:

(9) $$T_{S}=\frac{P_{avg}Q_{abs}}{4\pi Rk_{Air}}+T_{0}$$

where $Q_{abs}=\frac{P_{abs}}{P_{inc}}=3.67\cdot 10^{-4}$ is the absorptivity of the particle's material. Thus, for 50 mW laser power used in the experiment, the particle is heated to a temperature of ≈60 °C. This number is verified by the experiment (50 mW and 60 °C are on top of each other in Figure 6b). Additional derivation can be found in Sections S3 and S4 (Supporting Information).

### 2.6 Ballistic Experiment

#### 2.6.1 Ballistic Target

For a more accurate assessment of the particle's velocity and with an outlook on needle‐free injection applications, the particle was jumped against a ballistic target. Using a setup similar to that in Figure 2, we jumped Rhodamine‐B‐infused vaterite particles from a glass substrate upwards into an SU8 layer. The layer was positioned several millimeters above the sample. We then located the jumped particles in the layer using a confocal fluorescent microscope, marked their location, and then imaged the sample with SEM. Residuals of the particle with the SU8 layer can be seen in Figure 6b. By assessing the depth of the crater within the polymer, the penetration depth of ≈400–600 nm can be deduced. To relate the results to a theoretical model, we used the Poncelet model,^[^ ^81^, ^82^ ^]^ and the maximum penetration depth is given by:

(10) $$z_{\max}=\frac{2}{3}\frac{\rho_{s}}{\rho_{f}}\frac{d}{C_{D}}\ln\left[\frac{\rho_{f}C_{D}u_{0}^{2}}{2\gamma }+1\right]$$

where γ ∼ 1[*MPa*]‐ constant yield resistance (strength resistance);$C_{D}\approx \frac{24}{Re}$ – drag coefficient; *u*_0_‐ the impact velocity; *d* = 4[µ*m*] – particle diameter;ρs/f=2500/1190[kg/kgm3m3] – density of particle/target;η_0_ ∼ 1[*mPa* · *s*] – dynamic viscosity;$Re=\frac{\rho_{f}u_{0}d}{\eta_{0}}$ – Reynolds number. The particle's velocity can be deduced by the penetration depth and the rest of the parameters. Substituting z_max_ = 400–600 nm, as obtained in the experiment (Figure 6c), an initial velocity is estimated as u_0_ = 15–20 m s^−1^. This result appears as a red dot in Figure 5 and corresponds well with the rest of the data, thus verifying that the key mechanism beyond the observed effect is indeed thermos‐optical.

#### 2.6.2 Biological Target

**Figure 7:**
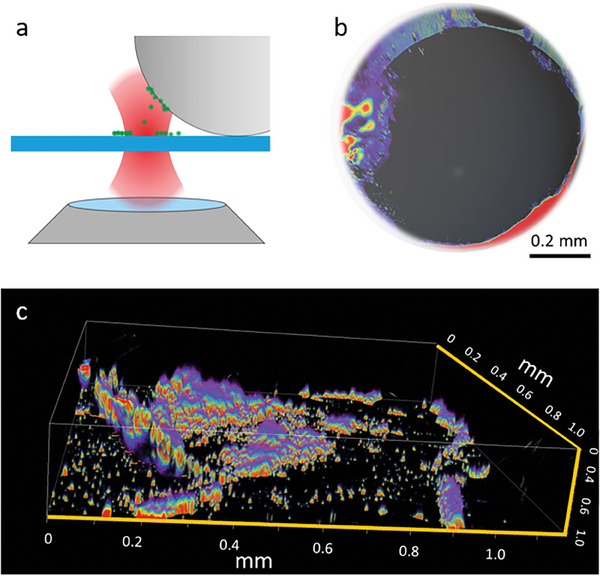
a) Schematic view of the ballistic experiment on *Xenopus oocyte*. b) 3D confocal image – combined bright field and fluorescence Z‐stack. c) 3D view (fluorescence Z‐stack) of the fluorescence after launching Vaterite fluorescent particle on the cell.

In‐vitro experiments were established using the new needle‐free injection method to simulate introducing of genetic material into Xenopus laevis oocytes, ensuring minimal damage to the cells. *Xenopus laevis* oocyte is a pre‐stage of a frog egg before its development.^[^ ^83^ ^]^ **Figure** **7**a presents a schematic illustration detailing the particle injection mechanism into the oocyte via the optical apparatus, visually demonstrating the nuanced and precise intervention technique that circumvents the conventional needle‐based approaches.

The result of multiple particles launching onto the oocyte appears in Figure 7b, demonstrating a combined 3D Z‐stack image, merging both a bright‐field optical image of the oocytes and a fluorescence Z‐stack post‐injection. The surrounding background (the glass coverslip) is removed from the image, which only encompasses the spherical oocytes. The biological sample itself is not fluorescent, only the particles loaded with Rhodamine dye can be observed (see Experimental Section). This result highlights the explicit delivery of fluorescent material into the oocyte, precisely at the side from which the particles were laser‐targeted. Note, that the oocyte itself is several millimeters across, thus making deep‐tissue fluorescent imaging difficult, and only areas close to the margins are clearly seen. Finally, Figure 7c provides a detailed 3D fluorescence image post‐injection, affirming the successful incorporation of the fluorescent particles within the oocyte. The other sides of the oocyte are not fluorescent, and the particles over the coverslip are also seen. As an outlook, for deeper penetration into the sample, the micron‐scale protein layer can be manually removed with a typical technique in the field.^[^ ^83^ ^]^ Three different oocyte samples were assessed, demonstrating similar performances, thus providing a signature of the reproducibility underscoring the potential of this methodology for advancing non‐invasive, targeted gene delivery, thereby opening avenues for refined electrophysiological explorations in future research endeavors.

## 3 Conclusion

Light‐driven tools can significantly contribute to the personalized medicine paradigm by granting drug delivery capsules with extra functions. Here we explored the possibility to realize a needle‐free injection scheme by accelerating a particle toward a target. Vaterite nanoparticles being among the most promising nonorganic drug delivery platforms have been explored. Apart from granting the particle sufficient velocity to penetrate the target, it has to be detached from a surface. To overcome a short‐range Van der Waals potential binding the capsule to the substrate without using excessive laser powers, femtosecond pulses have been used. After the observation of the particle jump effect, a physical model for the process has been proposed and analyzed. Optomechanical, Coulomb, and Shock Wave mechanisms were eliminated after the detailed analyses of the experimental data. The mechanism of fast picosecond‐scale optothermal expansion has been proposed, analyzed, and verified experimentally with the aid of nanothermometry tools, based on fluorescent lifetime imaging. Finally, the penetration capabilities of the particles were assessed by bombarding SU8 polymer layers. The Poncelet model was used to estimate particle velocities found in the 15m sec^−1^ range, given 0.5 W average power 100 fsec 1040 nm laser. Being relevant to biomedical applications, the demonstrated concept further strengthens the capabilities of optical tools in drug delivery applications.

## 4 Experimental Section

### Particle Acceleration Experiment

To assess the laser capabilities to accelerate nanoparticles, the following experiment was performed. Vaterite nanocapsules were placed on a glass coverslip and positioned on an inverted microscope (Figure 2a). A femtosecond laser (Menlo Systems, 100 fs pulse, 1040 nm wavelength, 100 MHz repetition rate) was launched through a half‐wave plate and polarizing beam splitter to control the light intensity illuminating the sample. To observe and track the release of the particles, a high‐speed camera (Phantom v7.1, maximal frame rate 110kfps) was used to capture the dynamics. The minimum speed of particles was estimated from the depth of field of the objective (≈0.8 µm) and the frame rate. Two dichroic mirrors were used to isolate the light source and camera from the laser.

In addition, a reference experiment was performed. The femtosecond laser was replaced by a CW source (Cobolt Rumba 1064 nm, 3 W) and the experiment was repeated, keeping the average power of the CW and femtosecond sources the same.

### Temperature Measurements

Fluorescence lifetime thermometry was used to determine the particle temperature. The time‐Correlated Single Photon Counting (TCSPC) method for measuring lifetimes was used. A super‐continuum laser (YSL SC‐PRO, 300 ps repetition rate, 450–530 nm filter, 3 mW average power to ensure not to induce an additional heating) was used as an excitation source of Rhodamine B. Vaterite particles were loaded with this dye by using a diffusion technique (e.g.,^[^ ^44^ ^]^). The femtosecond laser was also launched on the sample through a beam splitter, and its intensity was kept as a variable. Intensities were kept below the threshold, required for the jump (Figure 6a). The back‐scattered fluorescence was filtered and assessed with the photon counter. Reference measurements were done by varying the temperature of the sample with a thermo‐electric cooler. Ten different particles were measured to collect statistics and mitigate possible fluctuations.

### Maintenance and Operation of Xenopus laevis Frogs

Xenopus oocytes were kindly supplied by Prof. Nathan Dascal. Oocytes were obtainer frogs maintained and dissected as described in.^[^ ^84^ ^]^ Reconstitution of β‐adrenergic regulation of CaV1.2: Rad‐dependent and Rad‐independent protein kinase A mechanisms. Experiments were approved by Tel Aviv University Institutional Animal Care and Use Committee (IACUC permits # 01‐16‐104 and 01‐20‐083).

Oocytes were surgically extracted and defolliculated to ensure the eggs were isolated without surrounding follicular tissue. These frogs are widely utilized in scientific studies, particularly in developmental biology and physiology, due to their rapid development and translucent oocytes, which allow for easy observation under a microscope.

Vaterite particles were prepared and stained with Rhodamine B to facilitate fluorescent visualization. Initially, vaterite powder was vortexed for 2 h, followed by a 1 min sonication to ensure particle homogenization. Subsequently, the sample was washed meticulously with deionized water (DIW) and ethanol to remove any remaining impurities and excess dye. This meticulous preparation was vital for ensuring that the particles were suitable for subsequent interaction with biological samples, in this case, the Xenopus laevis oocytes.

The defolliculated oocytes were carefully extracted from the medium and positioned on a glass coverslip, upon which vaterite stained particles were drop casted. After the water had evaporated, leaving behind the stained particles, the oocyte was gently placed upon it. Utilizing a femtosecond laser within an optical tweezers apparatus, precise and controlled interactions with the oocytes were executed. Particularly, the laser was strategically targeted to one side of the cell, near its edge, minimizing potential damage and ensuring the integrity of the oocyte throughout the procedure.

Post‐interaction, the oocytes were swiftly transferred for confocal imaging to evaluate the interaction and infiltration of vaterite particles within the cellular matrix. This step was crucial to visualize the extent and pattern of vaterite particles' integration and to discern any immediate cellular responses to the intervention.

## Conflict of Interest

The authors declare no conflict of interest.

## Author Contributions

D.K., D.O., and A.M. contributed equally to this work.

## Supporting information

Supporting Information

Supplemental Video 1

Supplemental Video 2

Supplemental Video 3

## Data Availability

The data that support the findings of this study are openly available in Arxiv at https://doi.org/10.48550/arXiv.2305.11570, reference number 11570.
