# Supplementary material for: Optothermal Needle‐Free Injection of Vaterite Nanocapsules
Source: Adv Sci (Weinh). 2023 Dec 3;11(5):2305202. doi: 10.1002/advs.202305202 (PMC10837343; doi:10.1002/advs.202305202)
Supplement: Supplementary file 1 — Supporting Information [file ADVS-11-2305202-s001.pdf]

ADVANCED SCIENCE
Open Access

Supporting Information

for *Adv. Sci.*, DOI 10.1002/advs.202305202

Optothermal Needle-Free Injection of Vaterite Nanocapsules

Denis Kislov, Daniel Ofer, Andrey Machnev, Hani Barhom, Vjaceslavs Bobrovs, Alexander Shalin and Pavel Ginzburg*

SUPPORTING INFORMATION

Optothermal needle-free injection of vaterite nanocapsules

Denis Kislov, Daniel Ofer, Andrey Machnev, Hani Barhom, Vjaceslavs Bobrovs, Alexander Shalin, and Pavel Ginzburg.

S1. Calculating Hamaker constants

The Hamaker constant can be calculated using the Lifshitz approach [1]:

$$A_H \cong \frac{3}{4} k_B T \left(\frac{\varepsilon_1(0) - \varepsilon_3(0)}{\varepsilon_1(0) + \varepsilon_3(0)} \right) \left(\frac{\varepsilon_2(0) - \varepsilon_3(0)}{\varepsilon_2(0) + \varepsilon_3(0)} \right) + \frac{3h}{4\pi} \int_{\nu_1}^{\infty} \left(\frac{\varepsilon_1(i\nu) - \varepsilon_3(i\nu)}{\varepsilon_1(i\nu) + \varepsilon_3(i\nu)} \right) \left(\frac{\varepsilon_2(i\nu) - \varepsilon_3(i\nu)}{\varepsilon_2(i\nu) + \varepsilon_3(i\nu)} \right) d\nu \quad , \tag{S1}$$

where ν -frequency of the electromagnetic field; k_B - Boltzmann's constant; T - temperature in Kelvin; $\varepsilon_k(0), k = 1,2,3$ - static dielectric constant of media; $\varepsilon_k(i\nu), k = 1,2,3$ - dynamic dielectric constant of media (1-particle; 2-substrate; 3-environment); $\nu_1 = 2\pi k_B T / h = 3.9 \times 10^{13} Hz$ at $25^\circ C$.

Expression (S1) takes into account both the entropy ($\nu = 0$) and dispersion ($\nu > 0$) contributions, thus dielectric properties of the all three materials have to be known. Moreover, we can use the approximations of Ninham and Parsegian [2] and Hough and White [3] to calculate the constant. If the dielectric medium has one strong absorption peak at a certain frequency ν_e (the average ionization frequency of the material, most often in the ultraviolet region, typical value $\nu_e \approx 3 \cdot 10^{15} Hz$), the dielectric constant can be approximated by:

$$\varepsilon(i\nu) = 1 + \frac{n^2 - 1}{1 + \nu^2 / \nu_e^2} \quad , \tag{S2}$$

where n is the refractive index. Considering materials with similar dispersion (e.g. transparent dielectrics), the undelayed Hamaker constant (Eq. S1) is approximated with:

$$A_H \approx \frac{3}{2} k_B T \left(\frac{\varepsilon_1(0) - \varepsilon_3(0)}{\varepsilon_1(0) + \varepsilon_3(0)} \right) \left(\frac{\varepsilon_2(0) - \varepsilon_3(0)}{\varepsilon_2(0) + \varepsilon_3(0)} \right) + \frac{3h\nu_e}{8\sqrt{2}} \frac{(n_1^2 - n_3^2)(n_2^2 - n_3^2)}{\sqrt{n_1^2 + n_3^2}\sqrt{n_2^2 + n_3^2}\left(\sqrt{n_1^2 + n_3^2} + \sqrt{n_2^2 + n_3^2}\right)} \tag{S3}$$

So, the main contribution to the Hamaker constant comes from the visible and UV spectral ranges.

Table S1. The parameters for calculating the Hamaker constant [4]:

	$\varepsilon(0)$	n
medium 1 vaterite ($CaCO_3$)	8.2	1.6
medium 2 (glass)	3.82	1.46
medium 3 (air)	1	1

For smooth vaterite particles, the Hamaker constant is $A_H^{Vat} \approx 9 \cdot 10^{-20} J$.

S2. Optomechanical interactions with a femtosecond laser pulse

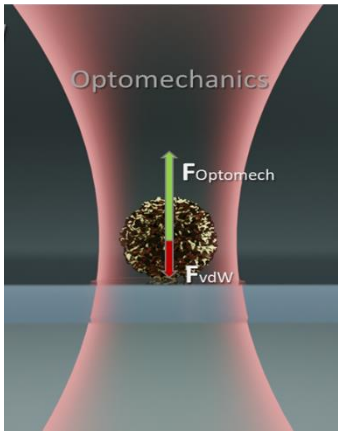

Figure S1 Sketch of the optomechanical interaction between a particle on a substrate and a laser pulse.

The temporal dynamics of optical forces can be assessed with the formalism of time-dependent Maxwell stress tensor. The electromagnetic optical force density is given by [5]:

$$f_i = \partial_j T_{ij} - \partial_t g_i \tag{S4}$$

The instantaneous force density f_i consists of two terms, both related to the transfer of momentum from the electromagnetic field to the particle. The first term corresponds to the time-dependent Maxwell stress tensor:

$$T_{ij} = E_i D_j + H_i B_j - \frac{1}{2}\delta_{ij}\left(E_k D_k + H_k B_k\right) \tag{S5}$$

The second term in Eq. S4 is the momentum carried by an electromagnetic field within the volume V. It originates from the dynamic terms in Maxwell's curl equations, containing the time derivatives:

$$g_i = \varepsilon_{ijk} D_j B_k \tag{S6}$$

Here ε_{ijk} is a completely antisymmetric Levi-Civita pseudotensor; E_i, D_i, H_i, B_i - Cartesian components of electric and magnetic fields and inductions.

To calculate the total optical force acting on a structure, Eq. S4 has to be integrated over the particle's volume. Applying Gauss theorem, we obtain [6]:

$$\mathbf{F}_{pulse}(t) = \frac{d\mathbf{p}}{dt} = \int_{\partial V} \overleftrightarrow{\mathbf{T}}(\mathbf{r},t)\cdot\mathbf{n}(\mathbf{r})\,ds - \frac{d\mathbf{p}_{field}}{dt}, \qquad \mathbf{p}_{field} = \frac{1}{c^2}\int_V [\mathbf{E}\times\mathbf{H}]\,dV \tag{S7}$$

Hereinafter, the momentum transfer to a particle illuminated with a single $\tau_0 = 100\,fs$ pulse centered at $\lambda_0 = 1040nm$ will be considered. Since the glass slide reflectivity is ~ 4%, the substrate can be neglected, and optical forces acting on an isolated particle (without the substrate) will be calculated. In the numerical setup, the particle is placed at the center of the Gaussian beam (radius $w_0 = 2\mu m$). In this case, only the radiation pressure will act on the particle, while the gradient force vanishes.

It should be noted that vaterite particles have anisotropic dielectric tensors. However, in [7], the internal structure of a spherulite was shown to demonstrate a moderately low impact on scattering patterns, i.e. the orientation of the particle with respect to the incident beam can be neglected for the estimation.

The dynamics of the momentum transfer to a particle can be calculated based on eq. (S7) as:

$$\mathbf{p}(t) = \int_0^t \mathbf{F}_{pulse}(t)\,dt \tag{S8}$$

Figure S5 demonstrates the time-dependent accumulated momentum for different average laser powers (in legends) and shows the result of the interaction of a short laser pulse with vaterite microparticles.

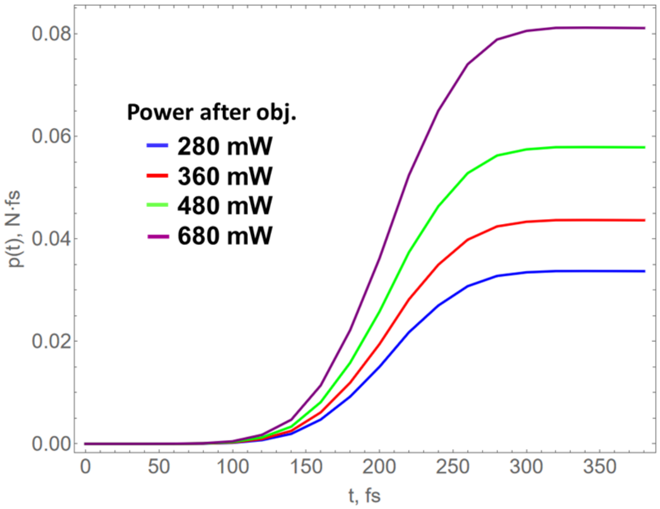

Figure S2. Dynamics of the mechanical moment transfer from a short laser pulse for vaterite particle ($n = 1.6$). Laser parameters: $\tau_0 = 100\,fs$ - pulse duration; $\lambda_0 = 1040nm$ - the laser wavelength; $w_0 = 2\mu m$ - the radius of the beam waist. Particle radius $R_0 = 2\mu m$.

Fig. S3 demonstrates the maximal optomechanical momentum as a function of the laser average power. The horizontal dashed line is a threshold momentum required for the jump (equivalent to

the van der Waals force attracting particles to the substrate) (see eq. 3 in the main text). Comparing the threshold momentum with the results suggests that particles cannot be detached from the surface if the primary physical mechanism is pure optomechanics.

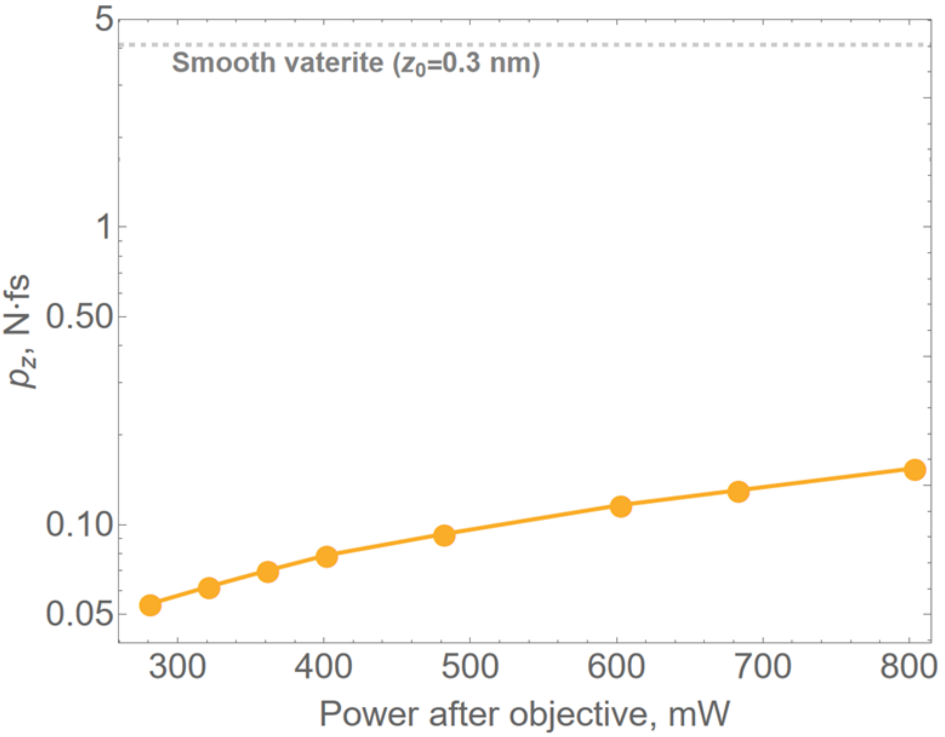

Figure S3. Momentum, transferred from a femtosecond laser pulse to a vaterite particle, as a function of the laser power. The dotted line indicates the threshold momentum.

S3. Vaterite Absorption

Vaterite microcapsules have relatively weak linear absorption [8]–[10]. The data was obtained by exploring spherical particles under CW illumination.

Table S2 – Experimental parameters from [9] and [10].

	**Parkin et. al.** [9]	**Dholakia et. al.** [10]
**Particle radius, µm**	1.6	0.423
**Wavelength, nm**	1064	532
**Beam waist, µm**	0.5	0.26
**Host media**	MetOH	D_2O
**Temperature (at 1 W), °C**	66	4.2
**Thermal conductivity, $Wm^{-1}K^{-1}$**	0.202	0.61

The imaginary part of the refractive index (n'') is responsible for heating. $n''_{1040} \sim 10^{-6}$, corresponding to calcite, was used for the estimations [11, 12]. Numerical analysis corresponding to parameters in Table S2 is summarized graphically in Fig. S4.

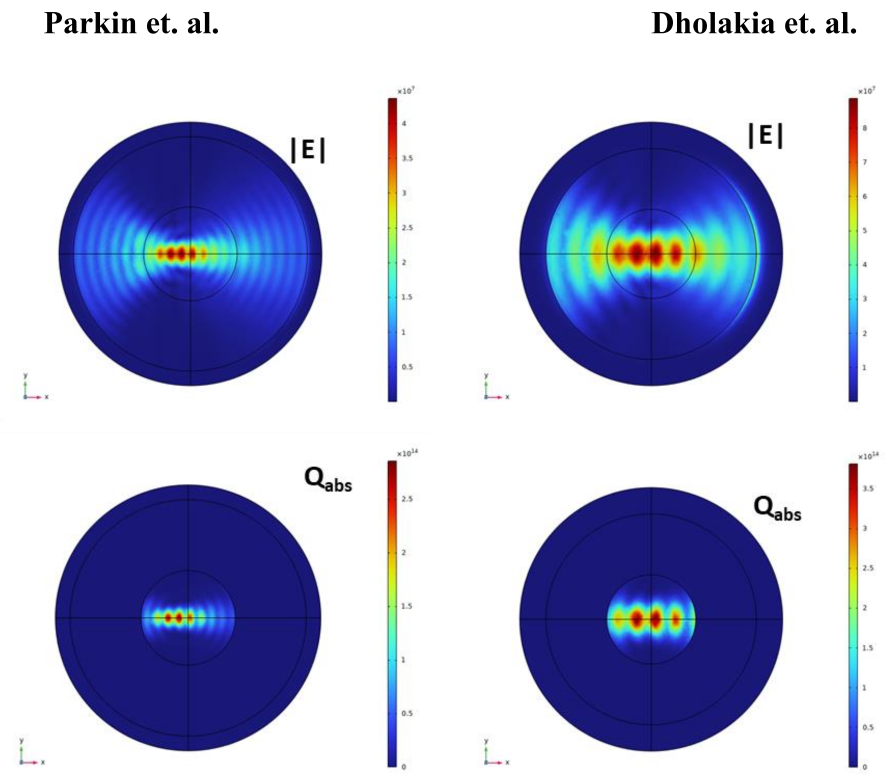

Figure S4. Electromagnetic interactions with microparticles. Details are in Table S2 and legends.

As a result of simulations, the following values were obtained: $n''^{(1064)}_{vat} = 8.5\cdot 10^{-6}$ and $n''^{(532)}_{vat} = 9.7\cdot 10^{-8}$. These values were used in the further analysis $n''^{(1040)}_{vat} \approx n''^{(1064)}_{vat} = 8.5\cdot 10^{-6}$.

The particle's temperature was also calculated analytically.

The model is based on a simple system of stationary heat diffusion equations in spherical coordinates:

$$\begin{cases} \frac{1}{r^2}\frac{d}{dr}\left(r^2\frac{dT_1}{dr}\right) = -\frac{q_0}{k_1} \quad inside\ NP \\ \frac{1}{r^2}\frac{d}{dr}\left(r^2\frac{dT_2}{dr}\right) = 0 \quad outside\ NP \end{cases} \tag{S9}$$

with boundary conditions

$$\begin{cases} T_1\big|_{r=R} = T_2\big|_{r=R} \\ T_1\big|_{r=0} < \infty \\ T_2\big|_{r\to\infty} = T_0 \\ -k_1 \left.\dfrac{dT_1}{dr}\right|_{r=R} = -k_2 \left.\dfrac{dT_2}{dr}\right|_{r=R} \end{cases} \tag{S10}$$

This system has the exact solution:

$$\begin{cases} T_1 = -\dfrac{q_0 r^2}{6k_1} + \dfrac{q_0 R^2}{3}\left(\dfrac{1}{k_2} + \dfrac{1}{2k_1}\right) + T_0 \\ T_2 = \dfrac{q_0 R^3}{3k_2}\dfrac{1}{r} + T_0 \end{cases} \tag{S11}$$

Then the temperature on the surface of a particle:

$$T_S\left(R\right) = \frac{q_0 R^2}{3k_2} + T_0 \text{ or } T_S\left(R\right) = \frac{P_{abs}}{4\pi k_2 R} + T_0 \tag{S12}$$

The absorbed power is given by:

$$P_{abs} = \frac{\omega}{2}\varepsilon_0 \operatorname{Im}\varepsilon \int_{V_{NP}} \left|\mathbf{E}\left(\mathbf{r}\right)\right|^2 dV \qquad \text{(S13).}$$

The absorptivity of the particle's material is defined as the ratio of the power absorbed in the particle to the power of the pulse $Q_{abs} = \dfrac{P_{abs}}{P_{inc}}$. To simplify the calculations, we assume the energy being absorbed uniformly $P_{abs} = q_0 V$, leading to $q_0 = \dfrac{3P_{abs}}{4\pi R^3}$. For CW laser $q_0 = \dfrac{3P_{avg}Q_{abs}}{4\pi R^3}$.

The simulation results are shown in the graphs below (Fig. S5). The solid curve is the analytical formula verified by the full-wave analysis (blue solid line).

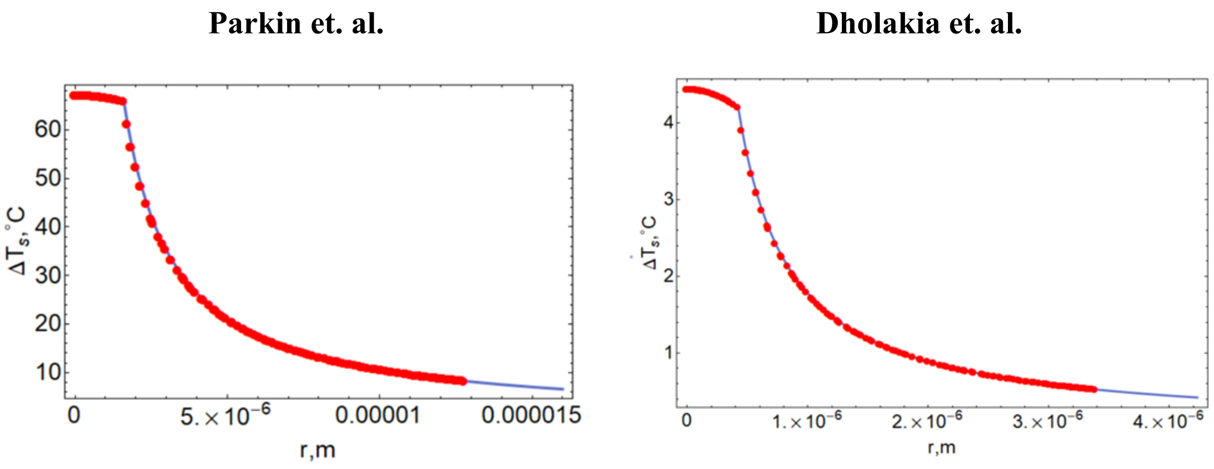

Figure S5. Temperature profiles for 1W laser power.

Temperature profile of a heated vaterite particle calculated based on the parameters in Table S3 and fitted values of the imaginary part of the complex refractive index. Red dots - modeling in COMSOL; solid lines are the spatial distribution of temperature, calculated by formula S11.

Further, using the obtained values of the imaginary part of the refractive index, a similar numerical simulation was carried out for a vaterite particle with parameters taken from our experiment.

Table S3 – Model parameters

**Particle radius, μm**	2	
**Wavelength, nm**	1040	
**Beam waist, μm**	2	
**Host media**	Air	H_2O
**Thermal conductivity, $Wm^{-1}K^{-1}$**	0.022	0.61

Numerical results demonstrating temperature profits at different conditions are summarized in Fig. S6.

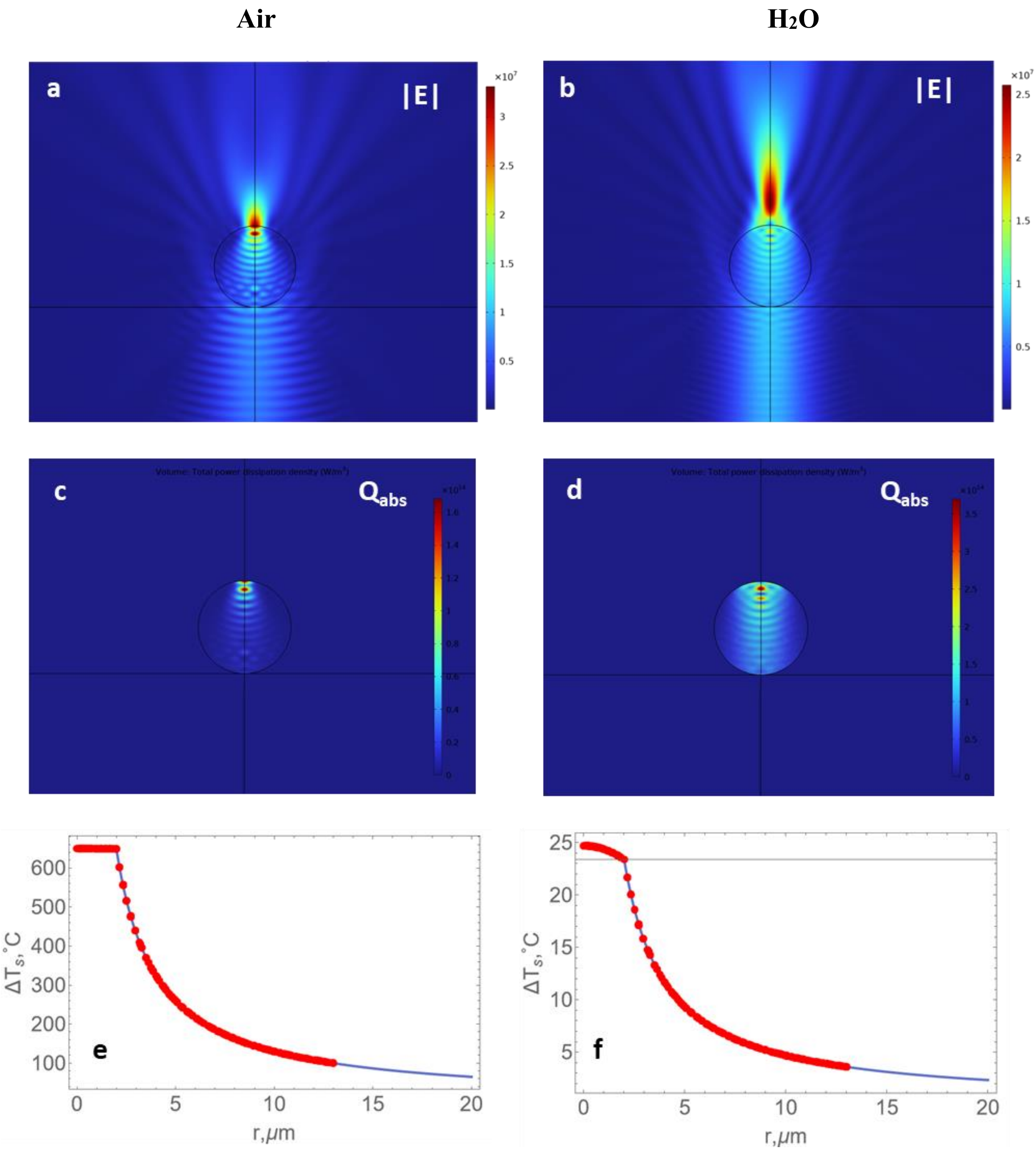

Figure S6. Results of numerical simulation. (a, b) Spatial distribution of the electric field for a vaterite particle located on a glass substrate. The system is irradiated with a linearly polarized Gaussian beam. The beam propagates from below. (c, d) Spatial distribution of the volume energy density absorbed by the particle (lossless substrate). (e, f) Temperature profile of a heated vaterite particle irradiated with a 1 W CW laser. Red dots - COMSOL; the solid line is the values obtained from formula S11.

S4. Pulsed laser heating of a particle.

In the case of short pulses, nonequilibrium phenomena govern the interaction. One temperature model is a mere approximation for the process [13]–[17]:

$$\begin{cases} \rho_l C_l \dfrac{\partial T_l}{\partial t} = k_l \dfrac{1}{r^2} \dfrac{\partial}{\partial r}\left(r^2 \dfrac{\partial T_l}{\partial r} \right) + q(r,t) \\ \rho_m C_m \dfrac{\partial T_m}{\partial t} = k_m \dfrac{1}{r^2} \dfrac{\partial}{\partial r}\left(r^2 \dfrac{\partial T_m}{\partial r} \right) \end{cases} , \tag{S14}$$

where T is the system's temperature, while subindexes m and l stay for an embedding medium and the object's lattice, respectively. C, ρ, and k are specific heat capacity, mass density, and heat conductivity. q(r,t) is the absorbed electromagnetic power density in the volume of a particle.

Boundary conditions are:

$$\begin{cases} \left.\dfrac{\partial T_l}{\partial r}\right|_{r\to 0} = 0 \\ T_l(R,t) = T_m(R,t) \\ T_m(\infty,t) = T_0 \\ T_l(0,t) < \infty \\ k_l \left.\dfrac{\partial T_l}{\partial r}\right|_{r=R} = k_m \left.\dfrac{\partial T_m}{\partial r}\right|_{r=R} \end{cases} . \tag{S15}$$

Initial conditions:

$$\begin{cases} T_l(r,0) = T_0 \\ T_m(r,0) = T_0 \end{cases} . \tag{S16}$$

Thermodynamic parameters for describing vaterite in air are:

$$\begin{cases} k_l = 5.5[W/m\cdot K] \\ \rho_l = 2500[kg/m^3] \\ C_l = 830[J/kg\cdot K] \end{cases} \quad \begin{cases} k_m = 0.022[W/m\cdot K] \\ \rho_m = 1.2[kg/m^3] \\ C_m = 1005[J/kg\cdot K] \end{cases} . \tag{S17}$$

The heat source resamples the form of the incident pulse:

$$q(r,t) = q_0 f(t) = q_0 \exp\left[-\frac{2(t-t_0)^2}{\tau^2}\right], \tag{S18}$$

where q_0 is a constant depending on the material absorption coefficient and the laser intensity.

Taking into account the relationship between the power in a pulse and the average energy and the time parameters of laser $P_{inc}^{pulse} = \frac{P_{avg}}{f_{rep}\tau}$, we obtain:

$$q_0 = \frac{3Q_{abs}P_{avg}}{4\pi R^3 f_{rep}\tau}. \tag{S19}$$

Figures S7 a and b show the results of the electromagnetic simulations. It can be seen that the field is focused by the particle formingt he so-called photonic nanojet. This leads to the creation of «hot spots". To simplify the analysis, we will approximate the process with a uniform distribution

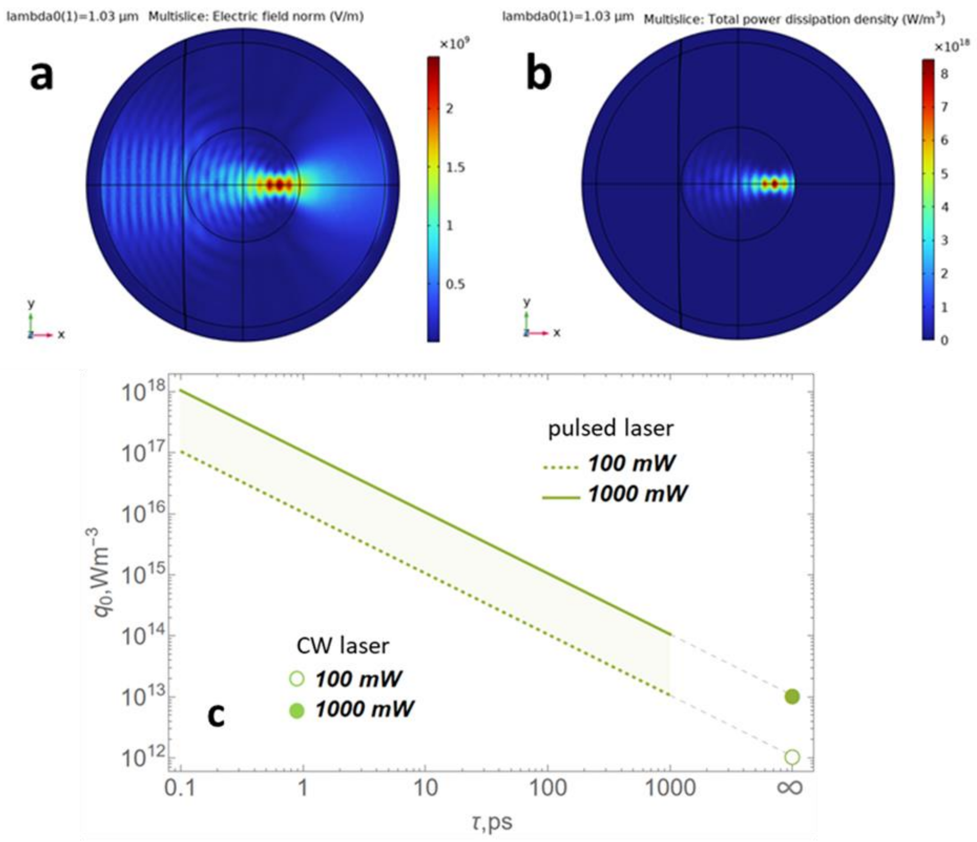

Figure S7. (a) electric field norm; (b) volume density of the absorbed electromagnetic power. (c) The value of q_0 constant as a function of the pulse duration for the average laser powers, ranging from 100 to 1000 mW. The dots show the value of the q_0 constant for a CW laser.

In this case, you can get an analytical solution:

$$T^{OTM}(t) = T_0 + \frac{\sqrt{\frac{\pi}{2}}\tau Erf\left[2\sqrt{2}\right]q_0}{2\rho C} + \frac{\sqrt{\frac{\pi}{2}}\tau Erf\left[\frac{\sqrt{2}(t-t_0)}{\tau}\right]q_0}{2\rho C} \quad \text{(S20)}$$

An analytical estimate of the temperature of a particle as a result of the absorption of a laser pulse of various intensities is shown in Figure S8. The calculations were performed for the parameters implemented in the experiment.

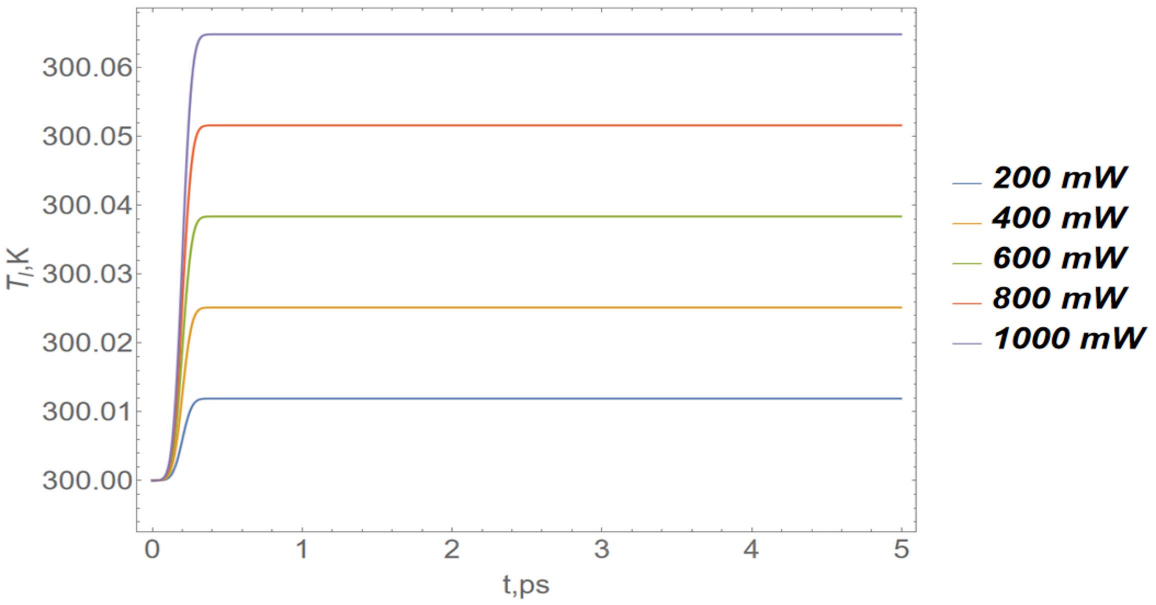

Figure S8. The time dependent temperature of a particle when irradiated with a laser pulse of different energies, calculated by formula (S20). Modeling parameters

$f_{rep} = 100MHz,\ w_0 = 2\mu m, R_{NP} = 2\mu m, T_0 = 300K, Q_{abs} = 3.67 \cdot 10^{-4}, \tau = 100\ fs$

S5. Influence of particle's size

Particle's size impacts three key factors:

1) The minimum impulse (speed) required to overcome the van der Waals adhesion force. The relationship between the minimum required speed and the particle radius is determined by equation (3) in the main text of the article.

$$u_{\min}(R_0) = \frac{1}{2R_0}\sqrt{\frac{A_H}{\pi z_0 \rho}} \tag{S21}$$

2) The momentum obtained by the particle due to thermal expansion resulting from its interaction with an ultrashort laser pulse is described by equation (7) in the main text of the article, wherein the value ΔT_{MAX}^{OTM} from equation (5) of the main text of the article is substituted.

$$u_z(R_0) = \frac{\alpha_{zz}^C \Delta T_{MAX}^{OTM} R_0}{\tau} = \frac{\alpha_{zz}^C}{\rho C} \cdot \underbrace{\frac{\sqrt{\pi}}{2\sqrt{2}}\left[1 + \mathrm{erf}\left[2\sqrt{2}\right]\right]}_{\approx 1.25} \cdot \frac{3Q_{abs}P_{avg}}{4\pi f_{rep}\tau} \cdot \frac{1}{R_0^2} \tag{S22}$$

3) The depth of penetration of an accelerated particle into a gel target (living tissue) is describe by equation (10) in the main text of the article.

$$z_{\max}\left(R_0\right)=\frac{2}{3}\frac{\rho_s}{\rho_f}\frac{d}{C_D}\ln\left[\frac{\rho_f C_D u_z^2\left(R_0\right)}{2\gamma}+1\right]=\frac{\rho_s}{9}\frac{R_0^2 u_z\left(R_0\right)}{24\eta_0}\ln\left[\frac{6\eta_0 u_z\left(R_0\right)}{\gamma R_0}+1\right] \tag{S23}$$

Further calculations were performed considering the material parameters and characteristics of the laser radiation that were implemented in the experiments presented in the article.

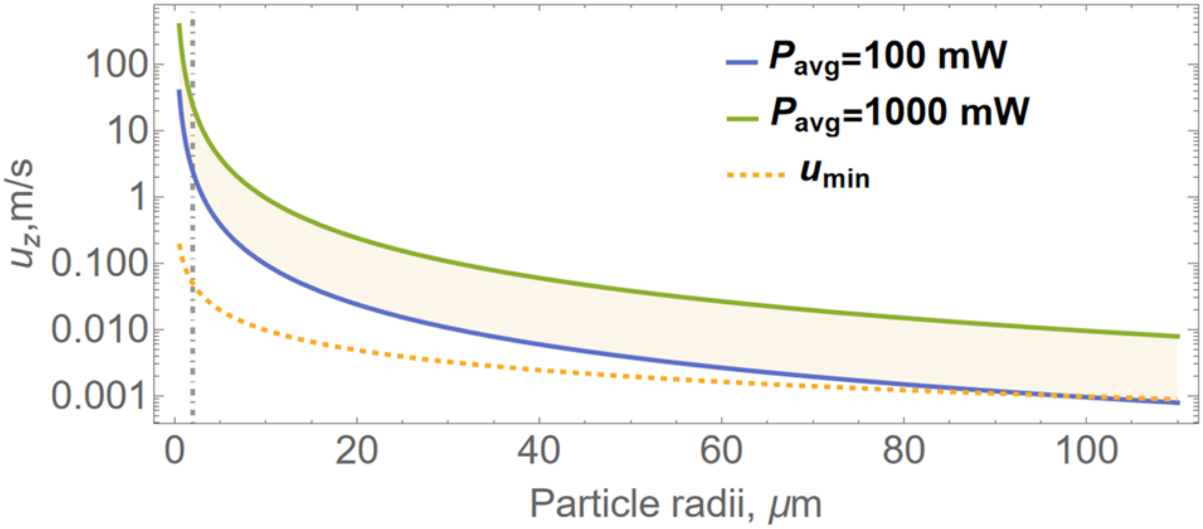

Figure S9. Dependence on the particle radius of the minimum required velocity (eq. S21) (dashed line) and the acquired velocity as a result of interaction with the laser pulse (eq. S22) (shaded area indicates the range of velocities for average laser power from 100 mW to 1 W). The vertical dashed line indicates the particle size used in our experiment.

From expressions (eq. S21) and (eq. S22) and the provided graph, it can be seen that for a large range of particle sizes (from tens of nanometers to several tens of micrometers), the acquired velocity resulting from interaction with the laser is higher than the minimum required velocity to overcome adhesion with the substrate. Thus, it can be concluded that for this range of particle sizes, particles will always detach from the substrate due to the mechanism described in the article. However, formally, at larger particle sizes (~100 μm), the acquired velocities are comparable, and with further increase in particle size, the acquired velocity becomes insufficient to overcome adhesion.

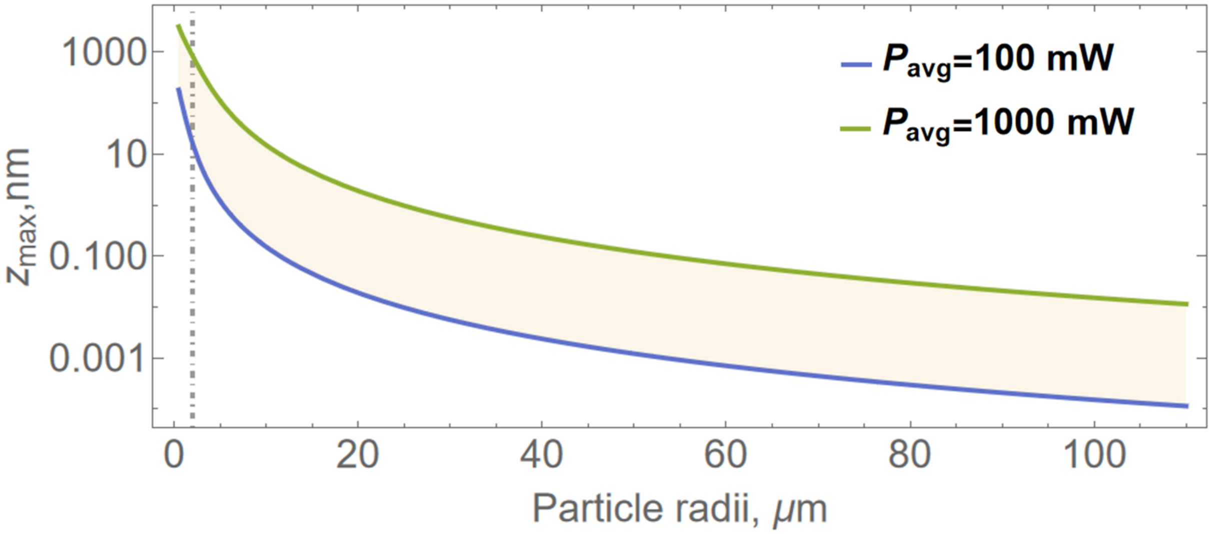

Figure S10. The dependence on the particle radius of the maximum penetration depth into the gel (tissue) target within the Poncelet model (eq. S23) (shaded area indicates the range of values for average laser power from 100 mW to 1 W). The vertical dashed line indicates the particle size used in our experiment.

It can be noted that practically significant penetration depths of vaterite particles into the target are achieved for particle sizes ranging from tens of nanometers to several microns.

It should be noted that when considering vaterite particles as containers for biologically active molecules, one of the most important characteristics is their size, which determines the possible method of their introduction into the body and the effectiveness of therapy. Studies show [18-20] that when vaterite is introduced into the body through injections or inhalations, particles with a size of less than 1 micron can easily penetrate and circulate in the bloodstream, including through small capillaries, and carriers with a size of 50-300 nm allow for the most effective penetration into cells. There is evidence that particles up to 100 nm, when administered intravenously, are able to penetrate the central nervous system through the blood-brain barrier.

Thus, it can be concluded that the chosen particle sizes of vaterite in our work are optimal both in terms of the effectiveness of detachment from the substrate using a laser pulse and in terms of the practical use of vaterite particles as containers for delivering biologically active molecules.

S6. The role of adhesion between particles on the driving force.

If the particles on the substrate are arranged in clusters, in addition to the adhesive forces between the substrate and the particle, there will also be adhesive forces between the particles.

The adhesive force for the "sphere-substrate" system can be expressed as follows:

$$F_{vdW}^{1}\left(z\right)=-\frac{A_H R}{6z^2} \qquad \text{(S24)}$$

For the "sphere-sphere" system with equal radii, the adhesive force within the framework of the Hamaker model can be expressed as:

$$F_{vdW}^{2}(z) = -\frac{A_H}{6z^2}\cdot\frac{R}{2} \tag{S25}$$

In this case, the first force acts normal to the substrate surface and provides the greatest resistance when accelerating the particle from the substrate. The second force is half as strong and acts parallel to the substrate.

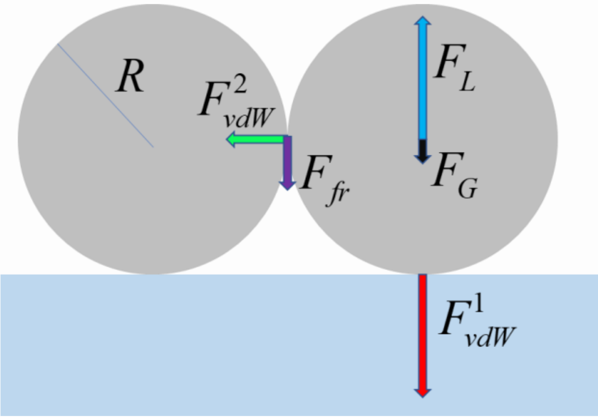

Fig.S11. Forces acting on a particle on a substrate in the case of cluster formation.

In this case, the van der Waals force between particles can be considered as the force that provides friction for particle sliding against each other. Accordingly, in order to achieve direct detachment of a particle from the substrate, the following condition must be satisfied for the lifting force (F_L):

$$F_L \geq F_{vdW}^{1} + F_G + F_{fr}$$

The gravitational force is much smaller than the force of adhesion $F_G \ll F_{vdW}^{1}$, and it can be neglected. The frictional force can be expressed as:

$$F_{fr} = \mu F_{vdW}^{2},$$

here μ is the coefficient of friction. For calcite powder, values in the range of 0.15-0.2 can be used [21]. Therefore, it can be written as:

$$F_L \geq F_{vdW}^{1} + F_{fr} = F_{vdW}^{1} + \mu F_{vdW}^{2} = F_{vdW}^{1} + 0.15\cdot 0.5\cdot F_{vdW}^{1} \approx 1.1F_{vdW}^{1}$$

Thus, in the case when clusters of particles form on the substrate, it is necessary to apply a force that is 10% greater than if the particles were individually distributed on the substrate in order to remove them from the surface.

The clustering can possess additional issues, but it can be prevented by functionalizing particles in and prevent aggregation in a solution. On a dry surface, the clustering can be prevented given a moderate concentration of particles.

1. E. M. Lifshitz, “The theory of molecular attractive forces between solids,” *J. Exp. Theor. Phys.*, vol. 2, no. 1, 1956.
2. Ninham, B. W., & Parsegian, V. A. (1970). van der Waals Forces across Triple-Layer Films. The Journal of Chemical Physics, 52(9).
3. Hough, D. B., & White, L. R. (1980). The calculation of hamaker constants from liftshitz theory with applications to wetting phenomena. Advances in Colloid and Interface Science, 14(1).
4. H. Butt, K. Graf, and M. Kappl, Physics and Chemistry of Interfaces. Wiley, 2003
5. J. C. Shane, M. Mazilu, W. M. Lee, and K. Dholakia, “Effect of pulse temporal shape on optical trapping and impulse transfer using ultrashort pulsed lasers,” *Opt. Express*, vol. 18, no. 7, 2010.
6. L. Novotny and B. Hecht, *Principles of Nano-Optics*, 2nd ed. Cambridge University Press, 2012.
7. H. Barhom *et al.*, “Biological Kerker Effect Boosts Light Collection Efficiency in Plants,” *Nano Lett.*, vol. 19, no. 10, 2019.
8. A. I. Bishop, T. A. Nieminen, N. R. Heckenberg, and H. Rubinsztein-Dunlop, “Optical microrheology using rotating laser-trapped particles,” *Phys Rev Lett*, vol. 92, no. 19, 2004, doi: 10.1103/PhysRevLett.92.198104.
9. S. J. Parkin, G. Knöner, T. A. Nieminen, N. R. Heckenberg, and H. Rubinsztein-Dunlop “Picoliter viscometry using optically rotated particles” Physical Review E **76**, 041507, 2007
10. Y. Arita, J. M. Richards, M. Mazilu, G. C. Spalding, S. E. Skelton Spesyvtseva, D. Craig, and K. Dholakia “Rotational Dynamics and Heating of Trapped Nanovaterite Particles” ACS Nano 2016, 10, 12, 11505–11510
11. Ted L. Roush “ Estimation of visible, near-, and mid-infrared complex refractive indices of calcite, dolomite, and magnesite” Icarus 354 (2021) 114056
12. https://www.astro.uni-jena.de/Laboratory/OCDB/carbonates.html
13. M. I. Tribelsky and Y. Fukumoto, “Laser heating of dielectric particles for medical and biological applications,” *Biomed Opt Express*, vol. 7, no. 7, 2016, doi: 10.1364/boe.7.002781.
14. V. K. Pustovalov, “Theoretical study of heating of spherical nanoparticle in media by short laser pulses,” *Chem Phys*, vol. 308, no. 1–2, 2005, doi: 10.1016/j.chemphys.2004.08.005.
15. C. N. Mihailescu, M. A. Mahmood, N. Mihailescu, and M. Oane, “One-Temperature Analytical Model for Femto-/Atto-Second Laser–Metals Drilling: A Novel Approach,” *Materials*, vol. 15, no. 14, p. 5010, Jul. 2022, doi: 10.3390/ma15145010.
16. G. Baffou and H. Rigneault, “Femtosecond-pulsed optical heating of gold nanoparticles,” *Phys Rev B Condens Matter Mater Phys*, vol. 84, no. 3, 2011, doi: 10.1103/PhysRevB.84.035415.
17. R. R. Letfullin, T. F. George, G. C. Duree, and B. M. Bollinger, “Ultrashort laser pulse heating of nanoparticles: Comparison of theoretical approaches,” *Advances in Optical Technologies*, 2008, doi: 10.1155/2008/251718.

18. Costa LMM, Olyveira GM, Salomão. Precipitated calcium carbonate nano-microparticles: applications in drug delivery. Adv Tissue Eng Regen Med Open Access. 2017;3(2):336–340
19. A. Vikulina, J. Webster, D. Voronin et al. Mesoporous additive-free vaterite CaCO3 crystals of untypical sizes: From submicron to Giant Materials and Design 197 (2021) 109220
20. D.B. Trushina, T.N. Borodina, S. Belyakov et al. Calcium carbonate vaterite particles for drug delivery: Advances and challenges Materials Today Advances 14 (2022) 10021
21. Sven Berg, Pär Marklund, Hans-Åke Häggblad, Pär Jonsén, Frictional behaviour of CaCO3 powder compacts, Powder Technology, Volume 228, 2012, Pages 429-434
